# United States Health Policies and Late-stage Breast and colorectal cancer diagnosis: Why such disparities by age?

**DOI:** 10.1186/s13561-015-0058-2

**Published:** 2015-07-15

**Authors:** Lee R Mobley, Tzy-Mey Kuo

**Affiliations:** 1 School of Public Health and Andrew Young School of Policy Studie, Georgia State University, Atlanta, GA USA; 2 Lineberger Cancer Center, University of North Carolina at Chapel Hill, Chapel Hill, North Carolina, USA

**Keywords:** Late-stage cancer diagnosis, Cancer control, Health market regulation, Age disparity

## Abstract

**Background:**

Colorectal and breast cancers are the second most common causes of cancer deaths in the US. Population cancer screening rates are suboptimal and many cancers are diagnosed at an advanced stage, which results in increased morbidity and mortality. Younger populations are more likely to be diagnosed at a later stage, and this age disparity is not well understood. We examine the associations between late-stage breast cancer (BC) and colorectal cancer (CRC) diagnoses and multilevel factors, focusing on individual state regulations of insurance and health practitioners, and interactions between such policies and age. We expect state-level regulations are significant predictors of the rates of late-stage diagnosis among younger adults.

**Methods:**

We included adults of all ages, with BC or CRC diagnosed between 2004 –2009, obtained from a newly available cancer population database covering 98 % of all known new cancer cases. We included personal characteristics, linked with a set of county and state-level predictors based on residence. We applied multilevel models to robustly examine differences in risk of late-stage cancer diagnosis across age groups (defined as age 65+ or < 65), focusing specifically on the effects of state regulatory factors and their interactions with age.

**Results:**

Late stage BC diagnoses range from 24 %-36 %, while CRC diagnoses range from 54 %-60 % of newly diagnosed BC or CRC cases across states. After controlling statistically for many confounding factors at three levels, age < 65 is the largest person-level predictor for CRC, while black race is the largest predictor for BC. State regulations of health markets exhibit significant interactions with age groups.

**Conclusions:**

The state regulatory climate is an important predictor of late-stage BC and CRC diagnoses, especially among people younger than Medicare eligible age (65). State regulations can enhance the climate of access for younger, less well-insured or uninsured persons who fall outside normative screening guidelines.

## Background

Colorectal cancer (CRC) is the third most common cancer in men and women, and is the second leading cause of cancer deaths in the United States. The risk of CRC begins to increase after the age of 40 years and increases more rapidly at ages 50 to 55 years. Overall, the risk doubles with each succeeding decade, and continues to rise exponentially [1]. The 5-year survival rate for CRC patients is about 65 %. Colorectal cancer incidence rates have been decreasing for most of the past two decades, which has largely been attributed to increases in the use of endoscopic colorectal cancer screening tests that allow the detection and removal of colorectal polyps before they progress to cancer. In contrast to the overall declines, among adults younger than 50 years, for whom screening is not routinely recommended, colorectal cancer incidence rates have been increasing each year since 1998 [2]. By comparison, breast cancer (BC) is the most frequently diagnosed cancer in women (excluding skin cancers), and ranks second as a cause of cancer death in women. The 5-year survival rate for BC patients is 89 %. The incidence rate for female breast cancer was rising until recently, due to common use of menopausal hormone therapy, but began to decline after publication of the landmark Women’s Health Initiative study in 2002 [3]. Since about 2003, breast cancer incidence rates have been fairly stable [2].

Progress has been made in the war on cancer, yet the high proportions of late-stage diagnoses remain a public health concern. The American Cancer Society states that only 39 % of CRC cases are diagnosed at an early stage [4], while about 80 % of BC cases are diagnosed at an early stage [5]. Thus about 60 % of CRC and 20 % of BC are diagnosed at a late stage.

National Cancer Institute statistics show that late-stage diagnosis rates are higher among younger populations than older populations for both BC and CRC [6]. The reasons for the disparity in late-stage cancer diagnoses by age are not well understood. Several studies have examined the predictors of stage at diagnosis for these two cancers, but have not focused on age as a predictive factor. Using SEER-Medicare data, studies have examined low SES, marital status, race or ethnicity, distance to closest provider, managed care penetration, area screening rates, or residence in a racially segregated community as predictors of late-stage diagnosis [7, 8]. Other studies which examined whole cancer populations in multiple states or regions in the US found factors such as area poverty or deprivation, lack of personal insurance, or percent uninsured in the area have associations with late-stage BC diagnosis [9, 10]. Another study found that patients privately insured or insured by Medicare plus supplemental plans had lower likelihood of being diagnosed at advanced stages of cancer than persons with other types of insurance, with highest rates among uninsured and Medicaid insureds [11].

In this study, we sought to gain a more comprehensive understanding of the predictors of late-stage cancer, focusing on age while controlling statistically for a large number of other factors. We use newly available data that are representative of the vast majority of the US to answer several new research questions, including: Holding constant environmental factors, do the odds of late-stage cancer diagnosis vary among people <65 and 65+, by cancer type (BC, CRC), and by state regulatory environment? Do contextual constructs in the patient’s residential environment predict their likelihood of late-stage diagnosis? Holding other factors constant statistically, are state-level regulations of health insurance or health professionals significant predictors of late-stage diagnosis?

## Methods

We examined cancer cases from the United States Cancer Statistics (USCS) database, which is a population-based surveillance system of cancer registries with data representing 98 % of the U.S. population. The database was developed by a joint effort by the Centers for Disease Control and Prevention (CDC) and the National Cancer Institute (NCI) to provide a single, pooled-state database of reconciled, comparable cancer information geocoded at the local level to facilitate cancer control planning and evaluation [12]. This comprehensive database is now available inside National Centers for Health Statistics (NCHS) and Census Research Data Centers (RDCs) to qualified researchers [13].

Most states participate in the USCS registry data system, but not all allow use of county of residence information (Illinois, Michigan, Missouri, Ohio). Three states did not participate at all over the timeframe of this study (Kansas, Maryland, Minnesota). We excluded these 7 states and an additional state, Virginia, because data were not available until 2007. We also excluded Alaska and Hawaii, because their county designations are much different than for mainland states (each island is a county in Hawaii, and Alaska has boroughs rather than counties), and data are missing for some county-level constructs. After exclusions, this resulted in 40 states being included in the analysis over the 2004–2009 period. County level data describing contextual characteristics of communities were obtained from the RTI Spatial Impact Factor Database [14], which derives from numerous sources. State-level data describing insurance and practitioner regulatory environments were downloaded from the National Conference of State Legislatures databases [15, 16]. Summarizing the conceptual model: multilevel factors including person, community, and state levels of influence are predictors of the odds of cancer diagnosed at an advanced stage. Data description, sources, and brief rationale for inclusion of each covariate are provided in Table 1.

**Table 1:** Multilevel Model Variables: Description, Rationale, Source, and Sample Statistics

Variable (units of measure)	Rationale for Inclusion	Source, date	BC Models		CRC Models	
Outcome: whether cancer patient was diagnosed at a late stage (regional or distant =1, else = 0)	Late stage diagnosis is indicative of lack of knowledge regarding personal cancer risk, or the importance or availability of screening; lack of timely or proximate access to services, lack of funds to pay for, and cultural or other barriers related to utilization of timely cancer screening.	SEER and NPCR cancer registry data made available through NCHS Research Data Centers, covering 2004–2009: http://www.cdc.gov/rdc	mean	sdev	mean	sdev
			0.308	0.461	0.543	0.498
Person-level predictors						
female (binary)	Only females are included in BC study. Although males do have BC incidence, the numbers are few. Both male and female are included in the CRC study, with male designated as the reference group.		1.000	0.000	0.487	0.500
black (binary)	The national statistics cite blacks as a disadvantaged group, with worse outcomes relative to whites, the reference group.		0.101	0.301	0.112	0.315
race all other (binary)	All other races and ethnicities were combined to make the model more parsimonious, relative to whites, the reference group. Includes 8 % Hispanic, 3 % Asian, 0.5 % Native American, 0.8 % other.	BC sample *n* = 981,457 CRC sample *n* = 558,568	0.126	0.332	0.124	0.329
age < 65 (binary)	Two age groups allow us to distinguish effects for the well-insured Medicare population from the less well-insured younger population with cancer, who may also be more genetically susceptible and more likely to be screened and diagnosed at late stage.		0.624	0.505	0.424	0.494
County-level predictors						
isolation black (index 0–1)	Isolation indices have been examined in a broad literature as contextual predictors of health behaviors and outcomes. At smaller geographic scales they are thought to represent social support, and at broader scales political clout. A higher index value represents a lower chance that minorities reside among whites, with a value of 1 indicating a perfectly segregated society (2000).	RTI Spatial Database (https://rtispatialdata.rti.org) which includes variables derived by authors under NIH funding and made publicly/freely available. Sources include Decennial Census 2000; CMS Geographic Area Service Files, 2005;100 % FFS Medicare claims extracts 2006; Census SAHIE 2005	0.257	0.214	0.259	0.217
isolation Asian (index 0–1)			0.072	0.086	0.068	0.085
isolation Hispanic (index 0–1)			0.216	0.203	0.209	0.205
managed care penetration (%)	Managed care has transformed the way medicine is practiced in highly-penetrated markets, with preventive care services more prevalent/utilized more intensively (2005).		15.9	14.7	15.3	14.7
Distance (miles)	Calculated as the average distance (miles) over all ZIP codes with centroid in the county to closest provider ZIP code. Greater distance to provider of BC (mammogram) or CRC (endoscopy) screening suggests impeded access to preventive care services. Based on 100 % FFS Medicare utilization of mammography or endoscopy services (2006).		6.02	6.10	5.15	4.80
Screening rate (%)	Percent of the 100 % FFS Medicare population residing in the county and alive all year that utilized cancer screening (mammography, endoscopy) (2006).		23.60	3.18	11.05	1.43
Percent uninsured (%)	% of the under-age-65 population who did not have health insurance (2005).		17.73	5.45	17.75	5.49
State-level Policy Variables						
Direct Access to Specialist (1 = yes, 0 = no) in 2004	Access to gastroenterologists, gynecologists or oncologists without need of referral from a primary care physician may result in better matching of patient/provider and more timely care. Hypothesized to increase access for less well insured individuals or those in more stringent managed care plans. Younger people tend to be enrolled in these plans, which are less costly but restrict access and choice. Source: NCSL, 2010.		0.956	0.206	0.951	0.216
Ban on Financial Incentives (1 = yes, 0 = no) in 2004	Not allowing insurers to reward physicians financially for substitution of lower cost care could result in use of more expensive cancer screening tests (e.g., colonoscopy vs sigmoidoscopy or FOBT), more accurate surveillance and better quality care. For BC screening, this law could impact prescribing the more expensive MRI breast exam recommended for denser breast tissue versus mammogram. Source: NCSL, 2010.		0.628	0.483	0.627	0.484
Greater Practice Latitude for Nurse Practitioners (1 = yes, 0 = no) in 2004	Allowing nurse practitioners the latitude to practice medicine in independent clinics, without physician supervision can improve access to primary care in underserved areas. Hypothesized to increase access to primary care providers, increasing the chance that a person will be encouraged to utilize cancer screening. Source: NCSL, 2013.		0.342	0.474	0.336	0.472

### Study population

We restrict the sample to adults of all ages with a first cancer diagnosis in 2004–2009. We excluded records when BC or CRC were not the primary cancers, records with unknown cancer stage or unstaged cancer, or when diagnosis was by autopsy (<1 % of all cases). For BC we excluded males. These restrictions yielded a CRC study population of 558,568 individuals and a BC study population of 981,457 individuals.

### Measures

Outcome variable. Using the SEER summary stage 2000 variable provided in the database, we coded regional or distant diagnosis as late stage, and *in situ* or localized diagnosis as early stage. We then created a binary indicator for each individual specifying whether their cancer was diagnosed at a late stage, or not.

County-level contextual variables. In the 40 states, there were 2,366 counties with outcomes data in the BC population and 2,361 counties with outcomes in the CRC population. We used the isolation index to measure residential segregation of minorities from whites, which has been shown to have social support or political influence effects in various studies [17, 18]. For measuring accessibility, in addition to average closest distance to provider in the county (based on 100 % FFS Medicare service flows) we included the 100 % FFS Medicare BC and CRC screening rates as robust county-level measures. We included managed care penetration to capture spillovers on area practice styles, following the recent literature which suggests there were significant managed care spillover effects on the utilization of endoscopy in 1999 [19] and its geographic availability during 2001–2005 [20]. Managed care penetration also captures urbanicity, so we included distance squared to help capture urbanicity and better isolate the effects of managed care. We included the percent of the under-age-65 population who had no health insurance.

Including person and area-level factors, we are better able to isolate the independent effects of age groups, defined as adults over/under age 65. The vast majority of individuals over age 65 are insured, while a lower proportion of younger individuals are insured. We do not know individuals’ insurance status, but we do control for area percent uninsured among the population aged <65. We interact age with state policy to determine whether the state policy effect is different among older and younger groups.

**Fig. 1:**
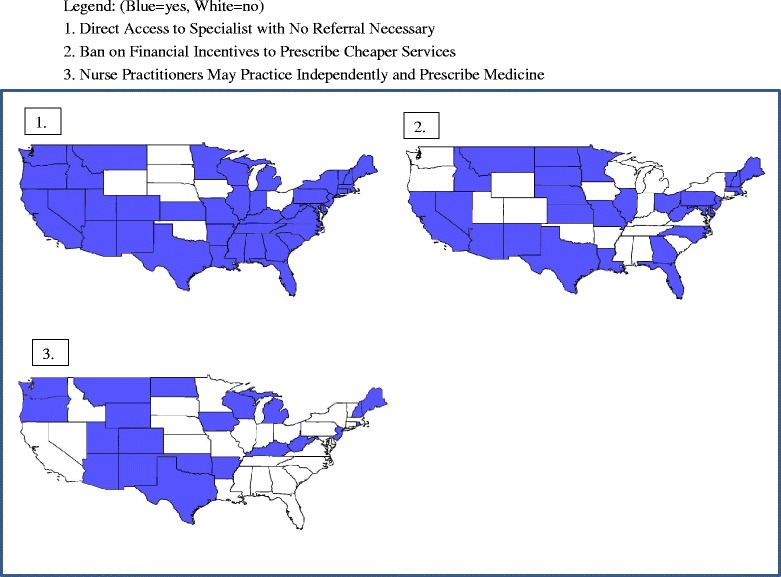
Three State Regulations in 2004. Legend: (Blue = yes, White = no). 1. Direct Access to Specialist with No Referral Necessary. 2. Ban on Financial Incentives to Prescribe Cheaper Services. 3. Nurse Practitioners May Practice Independently and Prescribe Medicine

State Regulations of Health Markets. We included three state policy variables depicted in Figure 1, summarized in Table 1. We hypothesize that these three state regulations could plausibly affect the availability of information regarding the importance of cancer screening, or the accessibility of specialists or physicians of choice to better align patients with the best medical advice. Ward et al. [11] found that people with the most preferred types of insurance were less likely to be diagnosed with late-stage cancer, while uninsured and Medicaid groups were more likely diagnosed at late stage. Thus timely access to preferred physicians and specialists seems important, and state regulations that enhance this may improve access; thus the first regulatory variable – Direct Access to Specialist – is expected to enhance early-stage diagnosis outcomes. For younger populations lacking insurance or enrolled in more restrictive insurance plans, this law would help circumvent gatekeeping activities that might delay timely advice. The Ban on Financial Incentives regulation is also expected to help patients receive the best quality care, by banning the practice by insurers of rewarding physicians financially for prescribing lower cost alternative services. This is expected to be especially important for CRC outcomes, where a lower cost alternative available and prescribed during this period – the fecal occult blood test (FOBT) – does not prevent cancer by removing lesions *in situ*, as do the endoscopy services. For BC outcomes, this practice would perhaps inhibit prescribing MRI for women with dense breasts in place of mammogram - which is cheaper, but perhaps less effective.

As regards nurse practitioner (NP) scope of practice laws - while physicians have traditionally been the main providers of primary care services, NPs are increasingly important in this area [21]. A systematic review of 26 studies published since 2000 found that health status, treatment practices, and prescribing behavior were consistent between NPs and physicians [22]. In areas with greater shortages of MDs, the NPs may serve a valuable role in providing primary care services and advice to encourage cancer screening and early detection. It remains an empirical question whether these NP practice scope regulations would have greater impact on the younger or the older population groups, which we investigate here.

### Statistical analysis

We used multilevel models to examine associations with late stage cancer diagnosis from predictors at person, county and state levels. We merged county-level community characteristics and state-level regulatory variables with person-level records from the USCS registry data system (Table 1).

We specify a three-level random intercept logit model for the late-stage diagnosis with patients nested in counties which are nested in states. We used a multilevel modeling (MLM) framework for estimation because we wanted to fit the regression to individuals while accounting for systematic, unexplained variation among counties and states. Ignoring the county and state level effects, when they are important, is tantamount to having omitted variables in the model, which can bias individual-level coefficient estimates. In addition, when the higher-level (e.g., county) covariates are of interest, failing to account for their structural similarity across individuals within the counties can yield biased standard errors for these county covariates, increasing their apparent statistical significance [23]. Both of these concerns led to the adoption of the three-level random intercept logistic regression estimated here.

The three-level model, with the cross-level interactions (age group and policy) across the person- and state- levels was estimated using GLLAMM, an add-on program to STATA (http://www.gllamm.org/). This software is the only software package we know of that can efficiently estimate a three-level random-intercepts model with a binary dependent variable, many groups (2,366 counties, 40 states) and a large sample size (1/2 – 1 million observations), [23, 24]. We estimated the logistic regression model of the binary cancer stage outcome separately for each cancer type and each state policy. The models for each cancer type used identical predictors, except CRC included an indicator to differentiate males and females.

## Results

Sample statistics, Table 1: For CRC, 54 % of cases have late stage diagnosis while for BC, 31 % of cases have late stage diagnosis. About half of the CRC sample are female. In both CRC and BC samples, the majority are white. The majority of the BC sample are younger than age 65 (62 %) whereas the opposite is true for the CRC sample (42 % younger than age 65).

Estimation results, Table 2: Overall, the person-level effects are quite similar across the policy models for each cancer type. The younger age group has significantly higher odds of late-stage diagnosis than those age 65+ in all models. Females have significantly higher odds of late-stage CRC diagnosis than males. Blacks, or other races or ethnicities (combined) have significantly higher odds of late-stage diagnoses than whites across all BC models, while only blacks have significantly higher odds of late-stage diagnoses than whites across the CRC models.

**Table 2:** Multilevel Modeling Results for Three State Regulatory Models: Predictors of Late-Stage Diagnosis of CRC

State Policy Variable:	Direct Access to Specialist				Ban on Financial Incentives				Greater Practice Latitude NP			
	odds ratio	*p*-value	Lower 95 % CI	Upper 95 % CI	odds ratio	*p*-value	Lower 95 % CI	Upper 95 % CI	odds ratio	*p*-value	Lower 95 % CI	Upper 95 % CI
Person level												
Age < 65	1.23	0.00	1.17	1.29	1.13	0.00	1.11	1.15	1.12	0.00	1.11	1.14
Female	1.05	0.00	1.04	1.06	1.05	0.00	1.04	1.06	1.05	0.00	1.04	1.06
Black	1.09	0.00	1.07	1.11	1.09	0.00	1.07	1.11	1.09	0.00	1.07	1.11
Race all other	1.01	0.31	0.99	1.03	1.01	0.44	0.99	1.03	1.01	0.37	0.99	1.03
County level												
<65 Pop uninsured	1.00	0.00	1.00	1.01	1.01	0.00	1.00	1.01	1.01	0.00	1.00	1.01
Distance to provider	1.01	0.00	1.00	1.01	1.00	0.38	1.00	1.01	1.00	0.51	1.00	1.01
Squared Distance	1.00	0.16	1.00	1.00	1.00	0.87	1.00	1.00	1.00	0.93	1.00	1.00
Screening rate	0.97	0.00	0.96	0.97	0.97	0.00	0.96	0.97	0.97	0.00	0.96	0.97
Managed care	1.12	0.01	1.04	1.21	1.02	0.64	0.93	1.12	1.06	0.20	0.97	1.17
Isolation black	0.85	0.00	0.81	0.89	0.98	0.61	0.93	1.05	0.99	0.83	0.93	1.06
Isolation Asian	1.64	0.00	1.34	1.99	1.15	0.05	1.00	1.32	1.25	0.00	1.09	1.43
Isolation Hispanic	1.04	0.28	0.97	1.10	1.05	0.24	0.97	1.14	1.01	0.78	0.93	1.10
State level												
State Policy	1.00	0.98	0.95	1.05	0.97	0.05	0.94	1.00	1.02	0.16	0.99	1.04
Cross level interaction												
Age <65* state policy	0.93	0.00	0.88	0.97	1.03	0.03	1.00	1.05	1.06	0.00	1.03	1.08
Random Intercept Parameters												
Level 1 variance*	3.2899				3.2899				3.2899			
Level 2 variance	0.0208				0.0220				0.0219			
Level 3 variance	0.0041				0.0068				0.0072			

*For logistic multilevel models, the variance for level one is assumed to be π^2^/3

County contextual variables. The percentage of the age <65 population who are uninsured is statistically significant at the 95 % level of confidence in 5 out of 6 models, but the effect estimate is very small. Area average distance to closest provider of screening services and its square do not have statistically significant effects in the models. The area level screening rate was positively associated with lower rate of late stage cancer diagnosis for both BC and CRC. In addition, the percent of the area population insured by managed care plans was associated with significantly lower odds of late-stage BC diagnosis, but significantly higher odds in one CRC model.

Higher values of the isolation index reflect greater isolation of the minority group from whites in their residential settings. The isolation index for Asians is associated with significantly higher odds of late stage CRC, and significantly lower odds of late-stage BC diagnosis, relative to cancer patients living in communities with low segregation of Asians. The isolation index for blacks is associated with significantly lower odds of late-stage CRC diagnosis, relative to cancer patients living in communities with low segregation of blacks, in the first CRC model only, and these are not significant predictors in the BC models. By contrast, living in more isolated Hispanic communities has no significant association with late-stage cancer diagnoses.

**Fig. 2:**
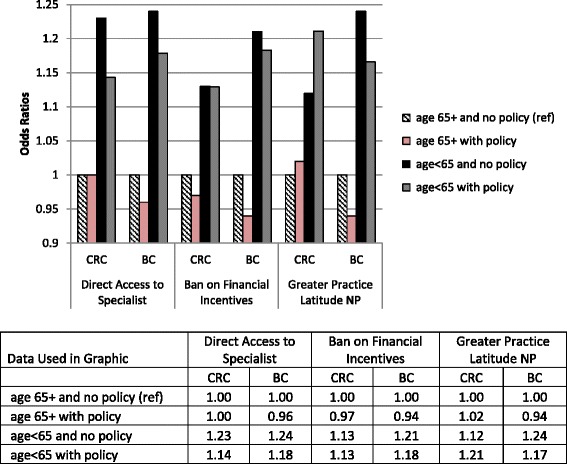
Odds Ratios for Late Stage Cancer Diagnosis: Age Group by State Policy Interaction, by Cancer Type

State insurance regulations. Our analyses found significant interactions in all CRC models and one BC model between age group and state policy, which is the main focus of the paper (Tables 2 and 3). The statistical significance of the age variable, state policy variables and their interactions with age group shown in Tables 2 and 3 cannot be interpreted separately, because the three variables are correlated by construction. Joint tests of the combined effects of age group and state policy and their interaction were statistically significant at greater than 95 % confidence for all three models and both cancer types (p val < 0.05 for joint test of coefficients). To better translate the associations between age groups and state policy, we computed odds ratios reflecting the different combinations of the two variables and plotted them in Fig. 2. The “age 65+ with no state policy in effect” is the reference group in the modeling, represented by the black-and-white striped bars’ height meeting the axis line at ‘1’ in the figure. The under age 65 group is represented by grey and black bars, while the age 65+ are represented by the striped and pink bars. The figure shows that for all three policies, younger people had higher late-stage diagnosis compared to older age groups, with or without policy. For the younger group, living in states where the first two policies were in effect seemed to help reduce the odds of late-stage diagnosis. For the older group, the first two policies also seem mildly beneficial (pink bars shorter than striped bars). For the third policy – greater practice latitude for NPs – for both age groups, the law seems beneficial for BC but not for CRC patients. Perhaps the advice from NPs regarding BC screening is more uncontroversial than such advice for CRC screening, which has serious associated medical risks from the endoscopy procedure.

**Table 3:** Multilevel Modeling Results for Three State Regulatory Models: Predictors of Late-Stage Diagnosis of BC

State Policy Variable:	Direct Access to Specialist				Ban on Financial Incentives				Greater Practice Latitude NP			
	odds ratio	p-value	Lower 95 % CI	Upper 95 % CI	odds ratio	*p*-value	Lower 95 % CI	Upper 95 % CI	odds ratio	*p*-value	Lower 95 % CI	Upper 95 % CI
Person level												
Age < 65	1.24	0.00	1.19	1.30	1.21	0.00	1.19	1.23	1.24	0.00	1.22	1.25
Black	1.46	0.00	1.44	1.48	1.46	0.00	1.44	1.48	1.46	0.00	1.44	1.49
Race all other	1.15	0.00	1.13	1.16	1.15	0.00	1.13	1.17	1.15	0.00	1.13	1.16
County level												
<65 Pop uninsured	1.00	0.02	1.00	1.00	1.00	0.01	1.00	1.00	1.00	0.34	1.00	1.00
Distance to provider	1.00	0.82	1.00	1.00	1.00	0.51	1.00	1.00	1.00	0.60	1.00	1.00
Squared Distance	1.00	1.00	1.00	1.00	1.00	0.86	1.00	1.00	1.00	0.89	1.00	1.00
Screening rate	0.98	0.00	0.97	0.98	0.97	0.00	0.97	0.98	0.98	0.00	0.97	0.98
Managed care	0.82	0.00	0.77	0.87	0.82	0.00	0.77	0.87	0.81	0.00	0.74	0.88
Isolation black	0.98	0.15	0.94	1.01	1.02	0.28	0.98	1.05	0.97	0.16	0.94	1.01
Isolation Asian	0.57	0.00	0.52	0.63	0.65	0.00	0.59	0.71	0.55	0.00	0.50	0.61
Isolation Hispanic	1.04	0.15	0.99	1.10	1.03	0.22	0.98	1.09	0.98	0.51	0.93	1.04
State level												
State Policy	0.96	0.08	0.92	1.00	0.94	0.00	0.91	0.96	0.94	0.00	0.91	0.98
Cross level interaction												
Age <65 * state policy	0.99	0.80	0.95	1.04	1.04	0.00	1.02	1.06	1.00	0.90	0.98	1.02
Random Intercept Parameters												
Level 1 variance*	3.2899				3.2899				3.2899			
Level 2 variance	0.0102248				0.01077366				0.01086441			
Level 3 variance	0.00314699				0.00563015				0.00280085			

*For logistic multilevel models, the variance for level one is assumed to be π^2^/3

The Direct Access to Specialist mandate is associated with the most dramatic reduction in the odds of late-stage cancers for younger people as compared to peers in unregulated states (e.g., for CRC, odds ratios are 1.23 vs 1.14 for the younger group in states without and with the mandate, respectively). Findings are similar for the Ban on Financial Incentives mandate, but effects are smaller. For both regulations, the younger group exhibits higher odds of late-stage diagnosis for both cancers, relative to the reference group/seniors, irrespective of policy status.

By contrast, living in states with greater practice latitude for NPs seems to benefit the younger group (1.24 falls to 1.17 in regulated states) and older age group (1.0 falls to 0.94 in regulated states) with BC. For CRC, this policy did not benefit either the younger group (1.12 no policy, 1.21 with policy) or the older group (1.0 no policy and 1.02 with policy).

Robustness: Because it is difficult to ascertain overlapping influences from the policies that may confound attribution of policy effects to particular policies, we ran a combined model. Combining the three policy variables together into a single model did not diminish the statistical significance of the Direct Access to Specialist policy variable. However the Ban on Financial Incentives became statistically weaker and NP practice latitude lost statistical significance. However, the three were significant as a group, and had consistent associations with those noted above.

Estimation Diagnostics: To ascertain the importance of information available in the random intercepts, it is customary to look at the variance components estimated by the model. We find that the variance of the random intercepts at the county and state levels are very small (bottom rows Tables 2 and 3). Two fairly recent papers examining late-stage BC diagnosis in California used the random intercepts multilevel model and found similarly small variance components [25, 8]. We conclude, as they do, that a small variance estimate for the area-level random effects indicates that the contextual factors included in the model do a good job accounting for spatial heterogeneity in the explanatory factors.

## Discussion/conclusions

This paper provides a comprehensive snapshot across 40 states in the US, based on fully generalizable registry populations, to assess the importance of various predictors of late-stage BC and CRC at first diagnosis for the period 2004–2009. The proportion of late-stage diagnosis is quite a bit higher for CRC (54 %) than for BC (31 %) (Table 1). These findings highlight the value of a new data resource for comprehensive cancer control research [13].

Separate models are estimated for the person-level odds of late-stage diagnoses for BC or for CRC. For each cancer type, three models are run reflecting three separate state policy regulations. There are some striking similarities in the effect estimates for each cancer type, across the three regulatory models. Person-level effects are quite similar across the regulatory models, and younger age group consistently exhibits higher odds of late stage diagnosis. Women have consistently higher odds of late-stage diagnosis for CRC, as compared to men. A higher area screening rate (for BC, CRC) is associated with significantly lower odds of late-stage diagnosis for both BC and CRC (respectively).

Some interesting differences are apparent across the BC and CRC models. Compared to CRC, the overall incidence of late-stage BC diagnosis is lower, but the racial and ethnic disparities are greater. While blacks and other races or ethnicities (combined) have significantly higher odds of late-stage diagnosis for BC than whites, only blacks have significantly higher odds of late-stage diagnosis for CRC than whites. This is puzzling, and would be a fruitful topic of further research. Perhaps more precise targeting of harder-to-reach minority enclaves is warranted to reduce these disparities.

Other differences across the cancer types are at the community level of influence, where we find several significant factors that contribute to our understanding of these population health outcomes. The percent of the community population insured by managed care plans was associated with significantly lower odds of late-stage BC diagnosis, but was *not* associated with significantly lower odds of late-stage CRC diagnosis. This dichotomy may reflect spillover effects from managed care which were apparently stronger for BC than CRC screening modalities. This would be expected, given the fact that screening guidelines for CRC were evolving during this period, while BC screening guidelines were well established [20].

Another interesting difference from community factors across models is the impact of residential segregation. For individuals living in highly segregated Asian communities, odds of late-stage BC diagnosis are significantly lower (than for people not living in such communities) while the opposite is true for CRC. Although it is unclear why, these differences may offer some insights for local cancer control efforts, where targeting certain populations may be of interest.

As shown in Fig. 2, the odds of late-stage diagnosis are considerably higher for the younger (<65) than older group (65+), and consistently so across the three state policy variables. Significant interaction effects between state policy and age group suggest that living in states where two of these policies have been enacted is beneficial for both young and old, and both cancer types. For the third policy, benefits seem evident only for BC diagnoses in both age groups. For the first policy, Direct Access to Specialist without a referral, the benefits in terms of odds reduction are greater for the younger than for the older group.

People in the younger group fall outside normative screening guidelines, and develop cancers for reasons related to greater than average risk factors, including genetic risk. We surmise that the younger group may have a greater need for regulatory protection, as they may be more financially vulnerable, and readily available or affordable insurance coverage may have been less adequate or more restrictive during this period. While we hold constant statistically the area uninsured rate, and find it to be a significant area-level predictor, we cannot know the person’s insurance status. A recent study using a different data source has shown that personal insurance is important to protect against late-stage diagnosis, and that not all insurances are equal in this regard [11].

We staged this study to end in 2009 so that future research using the USCS database will be able to evaluate ecological effects stemming from the passage of various insurance regulations and their implementation, notably the Affordable Care Act of 2010 and its provisions as they were phased in over time. An interesting future research question would be whether the role of area uninsured becomes less important over time, and whether the young-old age discrepancy and interaction with state regulation becomes less pronounced. This is a fruitful area of future research and the newly available USCS database will be quite valuable in further explaining these disparities and how they are impacted by policy changes.

## Abbreviations

BC: Breast cancer
CRC: Colorectal cancer
CDC: Centers for disease control and prevention
FOBT: Fecal occult blood test
MLM: Multilevel model
NCI: National cancer institute
NCHS: National center for health statistics
NCSL: National council of state legislatures
MRI: Magnetic resonance imaging
MD: Medical doctor
NP: Nurse practitioner
SEER: Surveillance, epidemiology, and end results
USCS: US cancer statistics
